# Stomach Carcinoma

**Published:** 1915-10

**Authors:** 


### ABSTRACTS

---

**Stomach Carcinoma,** J. C. Bloodgood, Baltimore, J. A. M. A., June 19, 1915. The series studied consists of 184 cases at Johns Hopkins Hospital in the last 25 years. In 45, there was no operation, in 49 exploration only, in 41 gastro-enterostomy —total ''inoperable'' 134. Resections 49. Of the 49 operable cases, 3 have survived 5 years or longer and may be considered cured. The maximum possible percentage of cures—providing all living patients show no recurrence—is 20, that actually accomplished for 21 resections up to 1910, 10%. Dating the duration of the disease from the beginning of ''continuous symptoms'' and not considering vague histories of years of indigestion, previous ulcer, etc., the statistics are as follows: 1-3 months, 8 operable among 27 cases; 3-6 months, 7 among 30; 6 months-1 year, 8 among 37; 1-2 years, 6 among 27; 2-5 years, 9 among 30; over 5 years, 2 among 8; totals: inoperable 34, exploration only 46, gastro-enterostomy 39 (total ''inoperable'' 119) resection 40 (total cases whose duration was estimated 159). Of the three apparently cured cases (one referred by M. Hartwig of Buffalo died of other cause 7 years later and showed no evidence of return of cancer) the durations were 8, 11, 11 months, respectively.

It will be remembered that it is contrary to the precedent of this journal to abstract from the J. A. M. A. or other weekly American journals, not from any hostility but because we feel that every one of our subscribers should receive the first which includes extensive reviews of the others and it is our endeavor to present matter not duplicated in the libraries of our subscribers. In this instance, we have been especially asked to comment on this article. The small number of cases is, at first, surprising, even disappointing but, on second thought, favorable in itself. For example, the writer has been deterred from presenting statistics from private practice partly because of the feeling that the series possible would be insignificant. But almost any surgeon or internist could in 25 years collect notes of series large enough to be of great value—perhaps of more value than large series representing the work of different men and not susceptible of so careful analysis. For a prominent author representing a famous institution to present thus candidly, a modest number of cases, is in itself an incentive to others.

Perhaps the best point made by the author is his inclusion of explorations and gastro-enterostomies with inoperable cases, without explanation or qualification. In our experience, the majority of explorations in cancer have been entirely without justification. That is to say, they have been performed without due regard to antecedent history and medical methods of diagnosis. In fact, the time to explore is before the diagnosis is made so that a series of explorations in cancer is merely part of a much larger series including many other conditions. Gastro-enterostomy, in the majority of cases does not deserve to be considered as an operation but only as a vivisection. Even with water-tight pyloric obstruction, life can be prolonged for about 40 days and if the pylorus is even slightly patulous, medical treatment can, without immediate risk, keep the patient comfortable and relatively free from intoxication and cachexia, for quite as long a time as that to be expected after palliative operation—and without narcotics except toward the end. These statements, it should be admitted, are subject to individual exceptions which should be carefully studied jointly from the standpoint of internal medicine and of surgery.

The data as to operability in reference to duration of disease are especially valuable because they are disappointing and contrary to the optimism prevalent as to early cancer. It will be noted that the operable cases which had presented continuous symptoms for only 1-3 months were a trifle less than 30% of all seen at this period, that exactly 30% of the 2-5 year cases were operable, that the lowest percentage of operable cases (22%) occurred in the six months-1 year period and that the average for the whole series of 159 cases was 25%. Note also that all three of the cured cases (conforming to the requirement of surviving without evidence of recurrence for at least five years) came in the six months-1 year period. From one standpoint, these facts are encouraging: there is hope of cure in cases long enough under observation or with long enough history to enable a diagnosis to be made with reasonable probability; at least, such cases are not physically speaking inoperable. But how do they fit in with the slogans that cancer is originally a local, eradicable process; that death is due to neglect on the part of the original attendant; that we can get a 25% rate of cures in favorable cases; that the laity should be taught to report early and that if they do, they stand a good chance of cure? The 1-3 and 3-6 months sub-series numbered, respectively 27 and 30, with only 8 and 7 cases physically available for radical operation and not one of these cases was cured. Our own experience—approximately equal in the total—warrants the opinion that the statistics are not misleading on account of accidental bad luck in the occurrence of cases, but that they represent actual, average conditions.

The plain fact of the matter is that, so far as cancer is concerned, the next move is not that of the surgeon, in perfecting his operative skill, nor of the clinician in developing his diagnostic art, nor of the medical publicist, official or voluntary in educating the laity but of the pathologist. We do not know whether cancer is exogenic or endogenic in origin. We do not know how long it exists in a latent—not merely an unrecognizable—state. We have no specific test for its diagnosis except such as depend on processes developed after it has reached a dangerous stage and for the most part coincident with clinical signs and symptoms sufficient for a reasonably probable diagnosis, nor are such specific tests pathognomonic.

